# Effect of N95 Respirator on Oxygen and Carbon Dioxide Physiologic Response: A Systematic Review and Meta-Analysis

**DOI:** 10.3390/ijerph19148646

**Published:** 2022-07-15

**Authors:** Kampanat Wangsan, Ratana Sapbamrer, Wachiranun Sirikul, Jinjuta Panumasvivat, Vithawat Surawattanasakul, Pheerasak Assavanopakun

**Affiliations:** Department of Community Medicine, Faculty of Medicine Chiang Mai University, Chiang Mai 50200, Thailand; kampanat.w@cmu.ac.th (K.W.); jinjuta.p@cmu.ac.th (J.P.); vithawat.surawat@cmu.ac.th (V.S.); pheerasak.assava@cmu.ac.th (P.A.)

**Keywords:** N95, respirator, respiratory physiologic response, carbon dioxide, oxygen saturation, respiratory protection equipment, physical activity, physical workload

## Abstract

During the COVID-19 pandemic, N95 respirators were commonly used in many situations. Respiratory problems from prolonged use of respirators were discussed in many studies, which show varied results. From the inconclusive results, the current systematic review and meta-analysis discerned the effects of the N95 respirator by assessing the oxygen and carbon dioxide changes in both high- and low-to-moderate-intensity physical activities in a healthy population. Thirteen studies were identified for inclusion in the study. In high-intensity physical activities, our meta-analysis showed borderline lower oxygen saturation and higher carbon dioxide partial pressure, but oxygen saturation did not change in low-to-moderate physical activity. The use of N95 respirators could statistically affect the physiologic changes of carbon dioxide and oxygen in high-intensity physical activity among healthy participants, but this may not be clinically significant. Some users who have certain health conditions, such as respiratory problems, should be informed of the clinical symptoms related to hypercarbia and hypoxia for the early detection of adverse effects of N95 respirators.

## 1. Introduction

During the COVID-19 pandemic, respirators were increasingly used by healthcare workers to protect against infection. The N95 disposable filtration respirator was the most common filtering facepiece respirator, with a 95% filtration rate for particles less than 0.3 microns, which can protect against highly transmissible diseases such as tuberculosis, SARS, and COVID-19 [1,2]. N95 masks were not only used by healthcare workers, but were also used widely among the general population to protect from community infections in many situations, such as general work and outdoor exercise.

Although N95 or equally standard respirators were used to protect from infection, some reported adverse health effects, including skin problems, headaches, dry eye, and impaired cognition [3]. Respiratory problems from prolonged use of respirators were discussed in many studies, which show varied results. One study by Mapelli et al. reported that using respirators was safe, with no significant differences in oxygen saturation even during high-intensity exercise [4]. In contrast, a study by Pimenta et al. showed a significant drop in oxygen saturation and warned of the cardiorespiratory impact of using respirators [5], but these might be only statistically significant, since arterial hypoxemia can be induced by exercise without any clinical significance [6]. From the inconclusive results, a systematic review and meta-analysis should be conducted to conclude the effect of N95 or equal level respirators on the respiratory system during various physical activities. 

The systematic review and meta-analysis by Keely et al. demonstrated interesting outcomes during exercise with various types of facemasks, including N95, surgical, and cloth masks [7]. The study reported minimal impact to physiologic changes, including slightly increased end-tidal CO_2_, heart rate, and respiratory rate [7]. However, the study did not classify the intensity of the activity, which might affect different physiologic outcomes [7]. Oxygen and carbon dioxide physiologic changes are an important marker of respiratory effects [8]. Our systematic review and meta-analysis aimed to discern the effects of the N95 respirator by assessing the oxygen and carbon dioxide changes in both high- and low-to-moderate-intensity physical activities in a healthy population. The results of the current study might bring about safety use guidance for N95 or equal respirator users in the pandemic and other situations.

## 2. Materials and Methods

### 2.1. Searching Strategy

This study was conducted in accordance with the systematic literature review and meta-analysis reporting guidelines of the Preferred Reporting Items for Systematic Reviews and Meta-Analysis (PRISMA). The current study was registered under PROSPERO registration number CRD42022298131, 4 January 2022. Our team searched for relevant articles published in the following databases: Scopus, Web of Science, and PubMed, using the following key words: (oxygen* or carbon dioxide) and (face mask OR surgical mask OR respirator OR N95 OR mask) AND (worker* OR exercise*).

### 2.2. Inclusion Criteria

The included studies met the following criteria: (1) original article; (2) published as a full article; (3) published in a journal or thesis; (4) published from January 1965 to January 2022; (5) evaluated the effect of N95 or equal level respirators on oxygen and carbon dioxide; (6) involved working or exercise experiment; (7) written in English; (8) data reported as oxygen saturation and partial pressure of carbon dioxide. The exclusion criteria were as follows: (1) articles without variables of interest; (2) review articles or letters to the editor; (3) articles with unrelated information.

### 2.3. Data Extraction

The data were extracted from the articles by the name of the first author, year of publication, study design, number and gender of participants, type of respirators, type of workload, and type of oxygen saturation (baseline, after the workload of the respirator, and control) and partial pressure of carbon dioxide (baseline, after the workload of the respirator, and control). Two investigators extracted the data independently.

### 2.4. Quality Assessment

The quality analysis was performed by using the National Heart, Lung, and Blood Institute (NIH) tools: (1) the Quality Assessment of Controlled Intervention Studies and (2) the Quality Assessment Tool for Observational Cohort and Cross-Sectional Studies. https://www.nhlbi.nih.gov/health-topics/study-quality-assessment-tools (accessed on 3 March 2022). Each checklist tool consists of 14 items for assessing the quality of studies. Two reviewers (K.W. and J.P.) independently assessed the quality of reporting in each study. The reviewers rated studies to assess the risk of bias in each study due to defects in study design or execution. Ratings were given for a range of items included in each tool to judge each study, with the quality being categorized as “good,” “fair,” or “poor”. The first criterion, “good”, indicated the least amount of bias. The second criterion, “fair,” was susceptible to some bias, but the level was considered insufficient to invalidate the results. The final score of “poor” indicated a risk of study bias.

### 2.5. Statistical Analysis

The conditions of interest were low and high physical workloads. The outcomes of interest were oxygen and carbon dioxide saturation. From eligible studies, the mean and standard deviation (SD) of the oxygen saturation (%) and partial pressure of carbon dioxide (mmHg) were retrieved, and we calculated the mean difference (MD) with a 95% CI as a summary measure for the meta-analysis outcomes. To assess heterogeneity, we used the Cochran Q and I^2^ tests against each other. We determined heterogeneity using the value of I^2^. An I^2^ value of 25% indicated low heterogeneity, I^2^ values of 25–50% indicated moderate heterogeneity, and I^2^ values greater than 50% indicated high heterogeneity. The pooled estimates of the oxygen saturation and carbon dioxide pressure among the respirators and the control group, before and after exercise or work, were analyzed using a fixed-effect model, utilizing the inverse-variance method for low-heterogeneity outcomes, as well as a random-effect model using the restricted maximum likelihood (REML) method for moderate-to-high heterogeneity outcomes. Funnel plots, displaying the standard mean differences of individual studies on the horizontal axis and the standard error on the vertical axis, were used to detect potential bias from small-study effects (e.g., publication bias). All of the statistical tests were two-tailed, and *p* < 0.05 was used to denote statistical significance. All of the statistical analyses were performed using the STATA software package (Stata Corp. 2019. Stata Statistical Software: Release 16. College Station, TX, USA: Stata Corp LLC).

## 3. Results

### 3.1. Search Study

**Figure 1:**
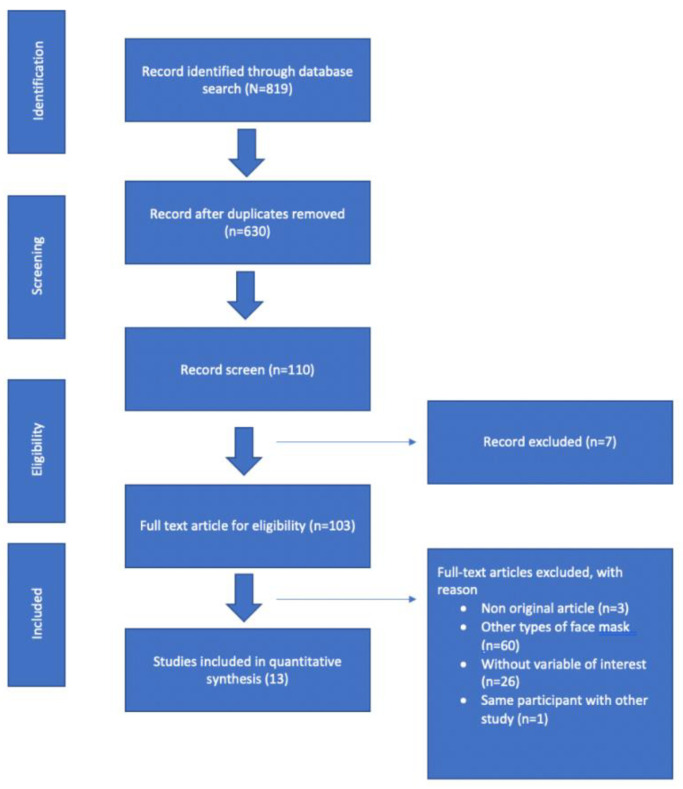
Flow Chart Study Selection Process (PRISMA). N = record identified through database searches; n = recorded data after searches.

The flow diagram in Figure 1 shows a summary of the method that was used. Our initial search of all of the databases retrieved 819 studies. After duplicates were removed, 630 articles remained, and 110 articles were screened based on the title and/or the abstract to determine eligibility. After screening, seven articles were excluded because there was no full-text paper. One hundred and one full-text articles were eligible. Ninety articles were excluded for the following reasons: (1) studies with no variables of interest; (2) other types of facemasks; (3) the same participants were used with other papers. Therefore, thirteen studies were included in the quantitative synthesis. 

### 3.2. Study Characteristics

Thirteen studies were identified for inclusion in the study. There were eight randomized cross-over studies, three non-randomized studies, and two observational studies. Twelve studies included information about oxygen saturation (Table 1 and Table 2), and eight studies included information about partial pressure of carbon dioxide (Table 3 and Table 4). Due to different physical workloads, the authors classified all studies following the American College of Sports Medicine’s (ACSM) guidelines for exercise testing and prescription into (1) high physical workload, such as high-intensity bench press and cardiopulmonary exercise until peak level or exhaustion by treadmill or ergometer, and (2) low-to-moderate physical intensity, such as low-intensity bench press, general healthcare work, or low-to-moderate speed on a treadmill or cycle ergometer [9]. The characteristics of the studies included in the meta-analysis are summarized in Table 1, Table 2, Table 3 and Table 4, including (1) oxygen saturation in a high physical workload, (2) oxygen saturation in a low physical workload, (3) partial pressure of carbon dioxide in a high physical workload, and (4) partial pressure of carbon dioxide in a low-to-moderate physical workload.

### 3.3. Differences in Oxygen Saturation Levels after a High Physical Workload

**Figure 2:**
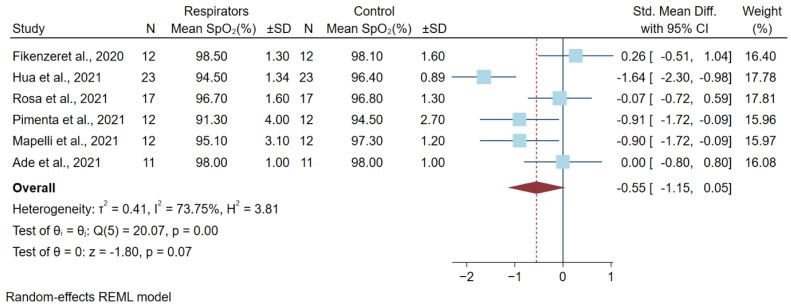
Difference in Oxygen Saturation Levels after a High Physical Workload. Abbreviations: CI, confidence interval; SD, standard deviation; SpO_2_, oxygen saturation level; Std. mean diff., standard mean difference [4,5,10,11,12,13].

Six studies described the oxygen saturation of high workload activity and included five randomized cross-over studies and one non-randomized study, with a total of 87 subjects. The respirator models in the studies were N95, FFP2, and KN95. Different types of physical workloads included high-intensity bench press, exercise on a treadmill or cycle ergometer, and incremental continued running tests. Five studies used transcutaneous oxygen saturation or a pulse oximeter (SpO_2_) to detect the oxygen saturation during the experiment. Only the study by Fikenzer et al., 2020 [13], reported oxygen pressure (PAO_2_) from blood gas analysis, which was converted to SpO_2_ by the equation of Brown et al. [21] before the meta-analysis. No studies showed evidence of fit testing (Table 1). All results of the mean difference of SpO_2_ between the respirators and the control group after the workload are shown in Figure 2. The pooled mean difference estimates found a statistically significant lower SpO2 in the respirator group for −0.55% [−1.15, 0.05].

### 3.4. Differences in Oxygen Saturation Levels after a Low-to-Moderate Physical Workload

**Figure 3:**
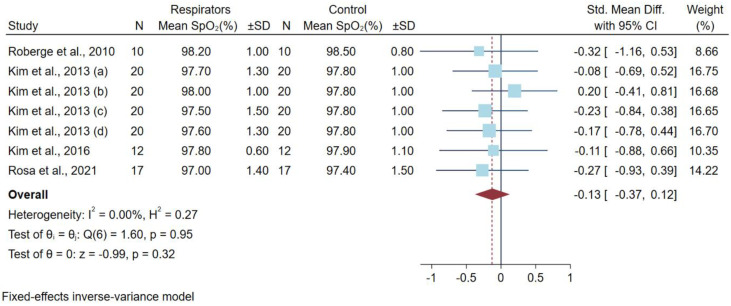
Differences in Oxygen Saturation Levels after a Low-to-Moderate Physical Workload. Abbreviations: CI, confidence interval; SD, standard deviation; SpO_2_, oxygen saturation level; Std. mean diff., standard mean difference [10,14,15,18].

Seven studies reported the SpO_2_ of low-to-moderate workload activity, including three randomized cross-over studies, two non-randomized studies, one prospective cohort, and one cross-sectional study. The study by Kim et al. [15] had four subgroup experiments. One hundred seventy-four participants were included. The respirators used in the studies were FFP2 and N95 respirators. The physical activities included in the studies were moderate-intensity bench press, treadmill exercise (at speeds of 4.02–5.6 km per hour), and healthcare work (210–240 min) (Table 2). All results of the mean differences of SpO_2_ between the respirators and the control group after a low-moderate workload are shown in Figure 3. The pooled mean difference estimates found no statistically significant lower SpO_2_ in the respirator group for −0.13% [−0.37, 0.12].

### 3.5. Differences in Partial Pressure of Carbon (PCO_2_) Dioxide after a High Physical Workload

**Figure 4:**
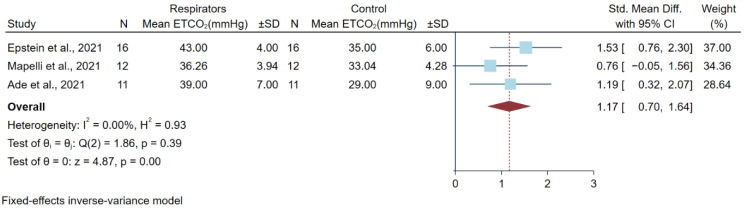
Differences in Carbon Dioxide Levels after a High Physical Workload. Abbreviations: CI, confidence interval; ETCO_2_, end-tidal carbon dioxide level; SD, standard deviation; Std. mean diff., standard mean difference [4,12,20].

Four studies reported PCO2 after a high physical workload. Three studies used end-tidal CO_2_ (EtCO_2_) to measure PCO_2_, and one study by Fikenzer et al. measured PCO_2_ using a blood gas analyzer [13]. A total of 51 participants were included, and all studies used a randomized cross-over design. The different respirator models included the KN95, FFP2, and N95 models. All studies used a cycle ergometer, with different protocols, as a physical workload. (Table 3). We could not extract the standard deviation from the study by Fikenzer et al. [13] due to missing data. Another three studies were analyzed in a meta-analysis. A meta-analysis from pooled different means of PCO_2_ after a high physical workload showed the statistical significance of higher PCO_2_ levels in the respirator group for 1.17 mmHg (0.70, 1.64) (Figure 4).

### 3.6. Differences in Carbon Dioxide Levels after a Low-to-Moderate Physical Workload

**Figure 5:**
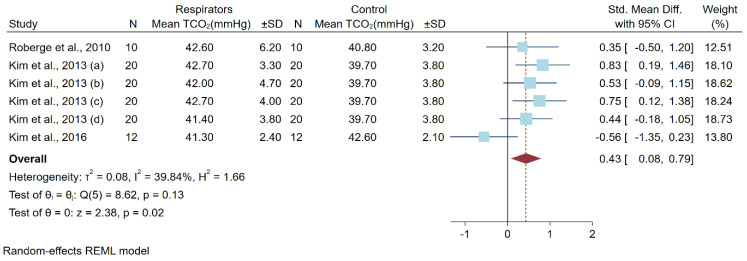
Differences in Carbon Dioxide Levels after a Low-to-Moderate Physical Workload. Abbreviations: CI, confidence interval; TCO_2_, transcutaneous carbon dioxide level; SD, standard deviation; std. mean diff., standard mean difference [14,15,18].

Four studies reported PCO_2_ after a low-to-moderate physical workload with 54 participants. The study by Kim et al. [15] had four subgroup experiments. All studies used transcutaneous CO_2_ (TcCO_2_) to measure PCO_2_. The study designs included two cross-over randomized controlled trial designs and two non-randomized controlled trial designs. All studies used N95-type respirators, but in different models. All studies’ physical workload was treadmill exercise, using a speed of 5.6 km per hour for one hour. Only a study by Powell et al. [17] had no fit testing (Table 4). A meta-analysis from the pooled different means of PCO_2_ after a high physical workload showed statistically significant higher PCO_2_ levels in the respirator group for 0.43 mm Hg (0.08, 0.79) (Figure 5).

### 3.7. Funnel Plots

**Figure 6:**
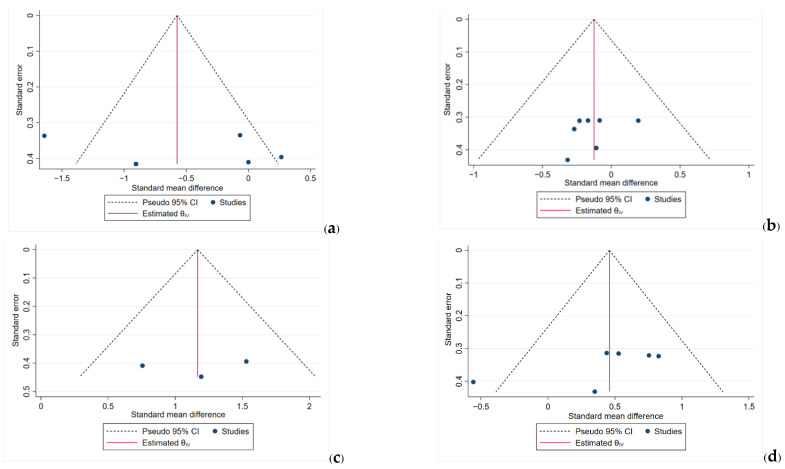
Funnel plots; (**a**) the differences in oxygen saturation after a high physical workload; (**b**) the differences in oxygen saturation after a low-to-moderate physical workload; (**c**) the differences in partial pressure carbon dioxide after a high physical workload; (**d**) the differences in partial pressure carbon dioxide after a low-to-moderate workload.

The funnel plots show symmetrical distribution patterns in Figure 6. There is an asymmetry pattern in Figure 6d; the different carbon dioxide levels after a low-to-moderate physical workload may be due to the small-study effect.

## 4. Discussion

The physiologic responses to oxygen and carbon dioxide might differ due to various factors, such as the type of respirators being worn by wearers who are under different conditions. In high-intensity physical activities, our meta-analysis showed borderline significant lower oxygen saturation and significant higher carbon dioxide partial pressure when using an N95 respirator. The breathing resistance while wearing an N95 respirator might explain the physiologic response. Breathing resistance depends on the type of respirator and the moisture inside the respirator. The temperature rises inside the N95 mask, bringing about the moisture from facial sweat and retained exhaled air. This moisture could block the respirator pores and increase breathing resistance [22]. One study by Heow et al. reported that the use of an N95 respirator demonstrated mean increments of 126 and 122% in inspiratory and expiratory flow resistances, respectively, and can cause hypoventilation from the reduction of 37% in air exchange volume [23]; in this study, the depletion of gas exchange led to the decrement of oxygenation and the accumulation of carbon dioxide production. 

Another factor affecting physiologic change was the carbon dioxide retention in a respirator’s dead space. In a normal atmosphere, the carbon dioxide volumetric concentration of ambient air is ∼0.03%, while in exhaled air, it is approximately 5%. Due to the porous resistance of the filtering respirator, the exhaled airflow with mixed CO_2_, in one breathing cycle, is confined in the residual dead space and is re-breathed in the next inhalation process [24]. The more carbon dioxide retention, the less oxygen concentration in breathing air, which eventually led to oxygen saturation depletion in this study. 

Our analysis also found that wearing the respirator without a high physical intensity workload might not interfere with oxygen physiologic change, but minimally increase carbon dioxide pressure. The explanation was that the conditions under which the respirators were used also played an important role in physiologic change, especially in physical activity. The high physical activity increased metabolic demand, with bodies requiring more oxygen consumption. When wearing a respirator under high physical activity conditions, the body may not be able to increase oxygen levels to meet the metabolic demands, because the resistance of the respirator might limit the air exchange and lead to the decrement of oxygen levels [25]. Blood lactate is also produced in high physical activity, which increases blood carbon dioxide and decreases blood oxygen. The more blood carbon dioxide contributed, the more carbon dioxide retention in respiratory dead space, eventually leading to lower oxygen saturation and higher carbon dioxide pressure [25,26]. In another way, low-to-moderate physical activity might not increase metabolic demand, and it could not significantly change oxygen saturation. Interestingly, the study by Fikenzer et al. [13] showed, in contrast, that even with the resistance of the respirator, the metabolic parameters (pH, PCO_2_, PO_2_, and lactate) did not have significant change. Further systematic reviews and meta-analysis should be conducted, focusing directly on the effect of the resistance of the respirator and the metabolic response. 

Even our results suggested that high physical activity could affect oxygen and carbon dioxide physiologic changes, but this was still subclinical change. The pulse oximetry threshold for detecting hypoxia is less than 92% (Carboxy-hemoglobin < 2%) [27]. Almost all studies showed a much higher SpO_2_ than 92% (94.5–98%); only the study by Pimenta et al. [5] reported a value lower than 92% (91.3%). This might be due to some of the participants from their study [5] having previous respiratory problems. Nevertheless, we could not confirm that it is completely safe to use an N95 respirator with high-intensity activities, because all studies in our review demonstrated hypercarbia. Carbon dioxide pressure (PaCO_2_) from blood gas analysis over 45 mmHg is defined as hypercarbia, which is approximately equivalent to 40 mmHg transcutaneous CO2 (TcCO_2_) [28]. This might be only short-term and not clinically significant in healthy participants. Some users who have certain health conditions, such as respiratory problems, must be informed of the clinical symptoms related to hypercarbia and hypoxia for the early detection of adverse effects of N95 respirators. Oxygen and CO_2_ are not the only physiologic markers of the respirator effect. Other markers, such as blood pressure and heart rate, should also be of concern in the safe use of respirators; several studies have already addressed these parameters [7,13].

Another method to reduce the respiratory effects of respirators is the adjustment of breathing resistance. To reduce the effect of breathing resistance, many respirator standards were designed to minimize breathing resistance. According to the NIOSH-42 CFR84 standard of the National Institute of Occupational Safety and Health (NIOSH), the N95 respirator should have an inhalation resistance max pressure drop (a flow rate of 85 L per minute) equal to or less than 343 pascals, and that of exhalation equal to or less than 245 pascals [29].

## 5. Limitations

Different measurement tools were utilized in measuring carbon dioxide pressure, including end-tidal CO_2_ (EtCO_2_) and transcutaneous CO_2_ (TcCO_2_), but were analyzed separately in different categories of physical activities. This may affect the results. Other limitations in our study that could affect the interpretation include the different study designs, the various models of respirators, the duration of the experiment, and fit testing. This current systemic review and meta-analysis aimed to study only the acute response of carbon dioxide and oxygen in healthy participants. The effects of the long-term use of respirators, or their use in vulnerable groups, such as older people, patients with lung disease, or pregnant women, should be investigated further.

## 6. Conclusions

Short-term use of N95 respirators could affect the physiologic changes of CO_2_ and O_2_ in high-intensity physical activity among healthy participants. All users must be concerned regarding the health effects of respirators used in high-intensity activity such as vigorous exercise.

## Figures and Tables

**Table 1:** Oxygen Saturation in High Physical Workload. ^a^ after 1st set of exercises, ^b^ after 4th set of exercises, ^c^ after 1 min warm-up, and ^d^ SpO_2_ was calculated from PaO_2_.

Author	Population and Study Country	Study Design	Physical Load	Respirators	Outcome				Confounding
					Respirators		Control		
					Baseline	After a Workload	Baseline	After a Workload	
Rosa et al., 2021 [10]	17 male recreational weightlifters, Brazil	Cross-over randomized controlled trial	High-intensity bench press	FFP2/N95	96.1 ± 2.3 ^a^	96.7 ± 1.6 ^b^	97.1 ± 1.6 ^a^	96.8 ± 1.3 ^b^	Training, experience of participant, and no fit testing
Pimenta et al., 2021 [5]	12 professionals (8 men and 4 women) Portugal	Cross-over randomized controlled trial	Exercise testing followed Bruce treadmill protocol	KN95 (Gb2626-2006)	95.6 ± 2.0 ^c^	91.3 ± 4.0	96.0 ± 1.6 ^c^	94.5 ± 2.7	No fit testing, age, gender, physical activity, and habits
Mapelli et al., 2021 [4]	12 subjects (6 men and 6 women) Italy	Prospective, randomized, double-blind, and cross-over design	Cardiopulmonary exercise to the peak level by cycle ergometer	FFP2 (KN95)	96.9 ± 1.2	95.1 ± 3.1	97.2 ± 0.9	97.3 ± 1.2	No fit testing
Hua et al., 2021 [11]	23 participants (6 men and 17 women) China	Non-randomized controlled trial	Incremental continuous running test (ICRT) compared with no mask	N95	96.4 ± 1.41	94.5 ± 1.34	97.4 ± 0.78	96.4 ± 0.89	No fit testing
Ade et al., 2021 [12]	11 participants (5 men and 6 women) USA	Cross-over randomized controlled trial	Four incremental ramp exercise tests until exhaustion on a cycle ergometer	Vertical-fold N95	98.0 ± 1	98.0 ± 1	98.0 ± 1	98.0 ± 1	No fit testing
Fikenzer et al., 2020 [13]	12 men Germany	Cross-over randomized controlled trial	Incremental exertion test until exhaustion by cycle ergometer	FFP2/N95	98.7 ± 0.5 ^d^	98.5 ± 1.3 ^d^	98.3 ± 0.2 ^d^	98.1 ± 1.6 ^d^	No fit testing, but seal check was performed

**Table 2:** Oxygen Saturation in Low-Moderate Physical Workload.

Author	Population and Study Country	Study Design	Physical Load	Respirators	Outcome				Confounding
					Respirators		Control		
					Baseline	After a Workload	Baseline	After a Workload	
Rosa et al., 2021 [10]	17 male recreational weightlifters, Brazil	Cross-over randomized controlled trial	Moderate-intensity bench press exercise	FFp2/N95	95.6 ± 2.4 ^a^	97.0 ± 1.4 ^b^	97.0 ± 1.5 ^a^	97.4 ± 1.5 ^b^	Training experience of participant, no fit testing
Kim et al., 2016 [14]	12 male adults USA	Cross-over randomized controlled trial	1 h on treadmill speed 5.6 km/h	N95	98.2 ± 0.8	97.8 ± 0.6	98.1 ± 0.7	97.9 ± 1.1	
Kim et al., 2013 (a) [15]	20 young subjects (13 men and 7 women) USA	Non-randomized controlled trial	1 h on treadmill speed 5.6 km/h 0-degree incline	N95 Moldex 2200	98.4 ± 0.9	97.7 ± 1.3	98.1 ± 1.3	97.8 ± 1.0	
Kim et al., 2013 (b) [15]				N95 Moldex 2300	98.1 ± 1.3	98.0 ± 1.0			
Kim et al., 2013 (c) [15]				N95 3M 9210	97.9 ± 1.4	97.5 ± 1.5			
Kim et al., 2013 (d) [15]				N95 3M 9211	98.4 ± 0.9	97.6 ± 1.3			
Choudhury et al., 2020 [16]	75 health care workers (35 men and 40 women) India	Prospective cohort study	4 h work in ICU	N95	97.87 ± 1.17	97.73 ± 1.12	N/A	N/A	Testing environment
Powell et al., 2017 [17]	12 adults (6 women and 6 men) USA	Non-randomized controlled trial	1 h on treadmill speed 5.6 km/h 0-degree incline	N95	98.9 ± 0.7	98.8 ± 0.7	N//A	N/A	No fit testing
Roberge et al., 2010 [18]	10 healthcare workers (7 women and 3 men) USA	Cross-over randomized controlled trial	1 h on treadmill speed 2.5 mile/h (4.02 km·h)	N95	98.1 ± 1.2 ^c^	98.2 ± 1.0	98.5 ± 0.8	98.5 ± 0.8	
Nwosu et al., 2021 [19]	28 healthcare workers (15 men and 13 women) Nigeria	Cross-sectional	Intra-operation room, work average 210 min	N95 (various models)	97.9 ± 0.8	97.8 ± 0.8	-	-	No fit testing

^a^ after 1st set of exercises, ^b^ after 4th set of exercises, and ^c^ after 1-min warm-up.

**Table 3:** Partial Pressure of Carbon Dioxide in High Physical Workload.

Author	Population and Study Country	Study Design	Physical Load	Respirators	Outcome				Confounding
					Respirators		Control		
					Baseline	After a Workload	Baseline	After a Workload	
Mapelli et al., 2021 [4]	12 subjects (6 men and 6 women) Italy	Prospective, randomized, double-blind, and cross-over design	Cardiopulmonary exercise to the peak level by cycle ergometer	FFP2 (KN95)	36.85 ± 6.14 ^a^	36.26 ± 3.94 ^a^	35.6 ± 5.7 ^a^	33.04 ± 4.28 ^a^	No fit testing
Epstein et al., 2021 [20]	16 male adults Israel	Multiple cross-over, self-control trial	Ramp exercise tests until exhaustion on a cycle ergometer	N95	41 ± 3 ^a^	43 ± 4 ^a^	39 ± 2 ^a^	35 ± 6 ^a^	The resting time between each test, no fit testing
Ade et al., 2021 [12]	11 adults (5 men and 6 women) USA	Randomized cross-over study	Four incremental ramp exercise tests until exhaustion on a cycle ergometer	vertical-fold N95	36 ± 4 ^a^	39 ± 7 ^a^	29 ± 7 ^a^	29 ± 9 ^a^	No fit testing
Fikenzer et al., 2020 [13]	12 men Germany	Cross-over randomized controlled trial	Incremental exertion test until exhaustion by cycle ergometer	FFP2/N95	39.3 ± 2.2 ^b^	34.9 ^b^ (missing SD)	40.2 ± 3.4 ^b^	34.2 ± 3.8 ^b^	No fit testing, but seal check was performed

^a^ end-tidal CO_2_; ^b^ PCO_2_ from blood gas analyzer.

**Table 4:** Partial Pressure of Carbon Dioxide in Low-Moderate Physical Workload.

Author	Population and Study Country	Study Design	Physical Load	Respirators	Outcome				Confounding
					Respirators		Control		
					Baseline	After a Workload	Baseline	After a Workload	
Kim et al., 2016 [14]	12 male adults USA	Cross-over randomized controlled trial	1 h on treadmill speed 5.6 km/h	N95	39.3 ± 4.0 ^a^	41.3 ± 2.4 ^a^	41.2 ± 1.3 ^a^	42.6 ± 2.1^a^	
Kim et al., 2013 (a) [15]	20 young subjects (13 men and 7 women) USA	Non-randomized controlled trial	1 h on treadmill speed 5.6 km/h 0-degree incline	N95 Moldex 2200	98.4 ± 0.9 ^a^	97.7 ± 1.3 ^a^	39.0 ± 3.4 ^a^	39.7 ± 3.8 ^a^	
Kim et al., 2013 (b) [15]				N95 Moldex 2300	98.1 ± 1.3 ^a^	98.0 ± 1.0 ^a^			
Kim et al., 2013 (c) [15]				N95 3M 9210	97.9 ± 1.4 ^a^	97.5 ± 1.5 ^a^			
Kim et al., 2013 (d) [15]				N95 3M 9211	98.4 ± 0.9 ^a^	97.6 ± 1.3 ^a^			
Powell et al., 2017 [17].	12 adults (6 women and 6 men) USA	Non-randomized controlled trial	1 h on treadmill speed 5.6 km/h 0-degree incline	N95	36.8 ± 2.0 ^a^	38.0 ± 1.9 ^a^	-	-	No fit testing
Roberge et al., 2010 [18]	10 health care workers (7 women and 3 men) USA	Cross-over randomized controlled trial	1 h on treadmill speed 2.5 mile/h (4.02 km·h)	N95	39.7 ± 2.6 ^a,b^	42.6 ± 6.2 ^a^	40.8 ± 3.2 ^a^	40.8 ± 3.2 ^a^	

^a^ transcutaneous CO_2_; ^b^ after 1-min workload.

## Data Availability

Not applicable.
